# Relationship between Serum Ferritin Levels and Sarcopenia in Korean Females Aged 60 Years and Older Using the Fourth Korea National Health and Nutrition Examination Survey (KNHANES IV-2, 3), 2008–2009

**DOI:** 10.1371/journal.pone.0090105

**Published:** 2014-02-25

**Authors:** Tae Ho Kim, Hee-Jin Hwang, Sang-Hwan Kim

**Affiliations:** 1 Division of Endocrinology, Department of Internal Medicine, Kwandong University College of Medicine, Gangneung, Republic of Korea; 2 Department of Family Medicine, Kwandong University College of Medicine, Gangneung, Republic of Korea; 3 Geriatric Center and Department of Family Medicine, Myongji Hospital, Goyang, Republic of Korea; Innsbruck Medical University, Austria

## Abstract

**Context:**

It has been suggested that elevated serum ferritin is associated with several metabolic disorders. However, there is no reported study assessing any association between serum ferritin and sarcopenia despite the close relationship between sarcopenia and metabolic disorders.

**Objective:**

We investigated whether serum ferritin was associated with sarcopenia in older Koreans.

**Design and Setting:**

We conducted a cross-sectional study based on data acquired in the second and third years (2008–9) of the fourth Korean National Health and Nutrition Examination Survey.

**Participants:**

In total, 952 men (mean age 69.0 years) and 1,380 women (mean age 69.3 years) aged 60 years and older completed a body composition study using dual energy X-ray absorptiometry.

**Measurements:**

Serum ferritin levels were measured. Sarcopenia was defined as an appendicular skeletal mass as a percentage of body weight that was less than two standard deviations below the gender-specific mean for young adults.

**Results:**

Serum ferritin levels were lower in women than in men. Women with sarcopenia showed a higher level of serum ferritin than women without sarcopenia (men: without sarcopenia 115.7 ng/mL and with sarcopenia 134.4 ng/mL vs. women: without sarcopenia 70.7 ng/mL and with sarcopenia 85.4 ng/mL). The prevalence of sarcopenia increased as the tertile of serum ferritin increased. However, statistical significance was only seen in elderly women (1^st^ tertile 6.3%, 2^nd^ tertile 8.0%, 3^rd^ tertile 12.0%; p = 0.008). Without adjustment, compared with those in the lowest tertile of serum ferritin level, participants in the highest tertile had an odds ratio of 2.02 (95% confidence interval = 1.26–3.23) for sarcopenia in women. After adjusting for known risk factors, the OR for sarcopenia was 1.74 (95% CI = 1.02–2.97) in women. There was no statistically significant association between sarcopenia and serum ferritin tertiles in men.

**Conclusions:**

Elevated serum ferritin levels were associated with an increased prevalence of sarcopenia in women but not in men from a representative sample of elderly Koreans.

## Introduction

Loss of muscle mass, especially skeletal muscle mass, is an important age-related change in elderly people. The term ‘sarcopenia’ is used to describe progressive muscle mass losses associated with aging according to Rosenberg in 1989 [1], although many alternative definitions and criteria have been suggested to characterize ‘sarcopenia’. The reported prevalence of sarcopenia ranges from 7 to 24%, due to the lack of a generally accepted definition and heterogeneity within study populations [2].

The loss of muscle mass leads to decreased physical activity. Both reduced muscle mass and physical activity with age contribute to a decreased energy expenditure in elderly people. This decrease then promotes fat accumulation and obesity. Because muscle is the main tissue responsible for insulin mediated glucose disposal, sarcopenia and related conditions can then promote insulin resistance [3], type 2 diabetes [4], [5], dyslipidemia, hypertension and metabolic syndrome. Furthermore, sarcopenia is associated with of cardiovascular disease, independent of other cardiovascular risk factors [2].

Iron is one of the essential inorganic substance for normal body physiology. However, iron overload may be associated with adverse health outcomes [6]. Serum ferritin levels reflect iron stored in the body [7]. Several studies have demonstrated an association between serum ferritin levels and metabolic disorders. There was a positive association between serum ferritin levels and fasting serum glucose and insulin concentrations in Finnish men [8]. In several other studies, elevated serum ferritin levels were associated with insulin resistance [9], [10], metabolic syndrome [11], [12], type 2 diabetes [13], and cardiovascular disease [14], [15].

Although we do not understand the connection between serum ferritin and metabolic disorders, chronic inflammation and oxidative stress have been suggested to be a link between elevated serum ferritin levels and the development of metabolic disorders [16]. Under oxidative stress, elevated serum ferritin may contribute to cellular or tissue damage leading to insulin resistance [17], [18].

Similarly, chronic inflammation and oxidative stress are thought be important causes of the loss of muscle mass [19]. Several studies in rats have also reported the accumulation of iron in skeletal muscles with aging [20], [21]. These observations are similar to those of metabolic disorders such as diabetes and metabolic syndrome. However, there is no reported study showing any association between serum ferritin levels and sarcopenia. In this study, we examined a national representative sample of elderly South Koreans to investigate the association between serum ferritin levels and sarcopenia.

## Materials and Methods

### Study Participants and Database

This study was based on data acquired in the second and third years (2008–9) of the fourth Korean National Health and Nutrition Examination Survey (KNHANES IV). The KNHANES has been conducted periodically since 1998 to examine the general health and nutritional status of Koreans. KNHANES IV was a cross-sectional and nationally representative survey conducted by the Division of Chronic Disease Surveillance, Korea Centers for Disease Control and Prevention, from July 2007 to December 2009. The survey consists of a health interview survey, a health behavior survey, a nutrition survey, and a health examination. Data were collected via household interviews and by direct standardized physical examinations conducted in specially equipped mobile examination centers. Food intake was evaluated by a 24-h recall method. The sampling frame was developed based on the 2005 population and housing census in Korea. Household units were selected by a stratified multistage probability sampling design for the South Korean population. Approximately 260,000 primary sampling units, each of which containd ∼60 households, were included in KNHANES IV. From primary sampling units, 200 sampling units were selected randomly in the second and third years of the KNHANES IV. Finally, 25,250 individuals aged >1 year were sampled (12,528 in 2008 and 12,722 in 2009); these individuals represented 7,682 households in 200 districts (3,707 in 2008 and 3,975 in 2009). From the initial 25,250 individuals sampled, 20,277 participated in the survey (9,744 in 2008 and 10,533 in 2009), giving a response rate of 80.3% (77.8% in 2008 and 82.8% in 2009).

We identified 2,725 subjects aged ≥60 years who completed the body composition examination using dual energy X-ray absorptiometry (DXA; Discovery-W, Hologic, Inc., Waltham, MA, USA). We excluded individuals who had liver cirrhosis, chronic liver disease, or chronic renal disease (*n* = 59), who had known diagnoses of any malignancy (*n* = 152), whose serum liver enzyme activities were more than twice the normal range (ALT and AST; >80 IU/L; *n = *48), who had increased WBC counts (>10,000 cells/mL; *n* = 81), who had probable hemochromatosis, based on abnormal values of serum ferritin (>500 ng/mL; *n = *30), and who had received treatment for anemia within the last 3 months (*n = *33). Because some individuals met more than one exclusion criterion, the total number eligible for the analysis was 2,332 (952 men, 1,380 women).

Height, weight, and blood pressure were measured. Body mass index (BMI) was calculated by dividing the weight (kilograms) by the height squared (square meters). Waist circumference measurements were taken at the end of the normal expiration to the nearest 0.1 cm, measuring from the middle point between the lower border of the rib cage and the iliac crest at the mid-axillary line. Blood samples were obtained in the morning following an overnight fast. The serum concentration of glucose was measured using a Hitachi automatic analyzer 7600 (Tokyo, Japan). The serum ferritin and insulin levels were measured using immunoradiometric assays with a 1470 Wizard gamma-counter (Perkin-Elmer, Turku, Finland). All clinical analyses were performed by the Neodin Ministry of Health and Welfare. Homeostasis model assessment estimate of insulin resistance (HOMA-IR) was calculated as fasting insulin (µIU/mL) × fasting glucose (mmol/L)/22.5.

Participants were questioned about their average amount and frequency of alcohol consumption in the month preceding the interview. Alcohol drinking status was categorized as non-drinking, low-risk drinking, and high-risk drinking. High-risk drinking was defined as more than seven drinks (men) or five drinks (women) at a time and more than 2 days per week. Those who drink less than seven drinks (men) or five drinks (women) at a time or drink less than 2 days per week were considered to be low risk drinkers. Participants were grouped as current smokers, past smokers, and non-smokers. Family incomes were defined as low, moderate-low, moderate-high, and high. Education status was categorized as below elementary, middle school, high school, and college or above. Participants also reported whether they had comorbidities, including hypertension, hyperlipidemia, diabetes mellitus, coronary heart disease, stroke, arthritis, osteoporosis, back pain, chronic lung disease, chronic liver disease, renal failure, cancer, peptic ulcers, and depression. Physical activity was ascertained by asking participants how often they engaged in exercise each week including a Korean version of the international physical activity questionnaire (IPAQ). Vigorous physical activity (PA) was defined as 3 or more days of vigorous activity of at least 20 minutes per day. Moderate PA or walking PA was defined as 5 or more days of moderate-intensity activity or walking of at least 30 minutes per day. Short forms of IPAQ identify the frequency and duration of moderate and vigorous-intensity physical activity, walking physical activity, and inactivity during the past week (http://www.ipaq.ki.se). Using the Ainsworth et al. Compendium, an average MET score was derived for each type of activity [22]. The following values continued to be used for the analysis of IPAQ data: Walking = 3.3 METs, Moderate PA = 4.0 METs and Vigorous PA = 8.0 METs. Using these values, four continuous scores were defined as Walking MET-minutes/week = 3.3× walking minutes× walking days, Moderate MET-minutes/week = 4.0× moderate-intensity activity minutes× moderate days Vigorous MET-minutes/week = 8.0× vigorous-intensity activity minutes× vigorous-intensity days and total physical activity MET-minutes/week = sum of Walking+Moderate+Vigorous MET-minutes/week scores.

### Definition of Sarcopenia

Whole and regional body compositions were measured by using whole body dual energy X-ray absorptiometry (DXA; Discovery-W, Hologic, Inc., Waltham, MA, USA). Appendicular skeletal muscle mass (ASM) was calculated as the sum of skeletal muscles in the arms and legs, assuming that all non-fat and non-bone tissue was skeletal muscle. We defined sarcopenia using ASM as a percentage of body weight (ASM/Wt), modified from the report of Janssen et al [23]. To establish the cut-off value for sarcopenia, two standard deviations (SD) below the gender-specific normal mean for the younger reference group (2,606 subjects, aged 19–39) in KNHANES IV was used [24]. The values were 29.33% (ASM/Wt) in men and 23.06% (ASM/Wt) in women.

### Ethical Issues

Because the KNHANES IV survey data are publicly available (http://knhanes.cdc.go.kr/knhanes/), ethical approval was not required for this study. Prior to the survey, all participants were informed that they had been randomly chosen to participate in the KNHANES IV survey with the right to refuse to be involved in further analyses, and signed informed consents were obtained. The data we used from the KNHANES database were fully anonymized.

### Statistical Analysis

Data are expressed as means ± SD or percentages. The clinical characteristics of the study population were analyzed by an independent t-test for continuous variables and a chi-square test for categorical variables. All analyses were performed separately for elderly men and women, including calculation of gender-specific tertiles for serum ferritin levels. The chi-square test was used to compare the differences in the prevalence of sarcopenia using different methods among tertile groups of serum ferritin levels. The relationships between ASM percent and clinical variables were examined using Spearman correlation analysis. We calculated the odds ratios (ORs) and 95% confidence intervals (CI) for sarcopenia in tertile groups of serum ferritin using logistic regression models after adjusting for age, BMI, waist circumference, comorbidity, protein intake, physical activity, hemoglobin, WBC count, vitamin D, and HOMA-IR. Due to their skewed distributions, energy intake, physical activity and HOMA-IR values were logarithmically transformed. To estimate p for trends, the ferritin tertile group variable was regarded as a continuous variable in the trend analysis. All statistical results were based on two-sided tests. Data were analyzed using the SPSS software (ver. 15.0 for Windows; SPSS Inc., Chicago, IL, USA).

## Results

### 1. Clinical Characteristics of Subjects

**Figure 1:**
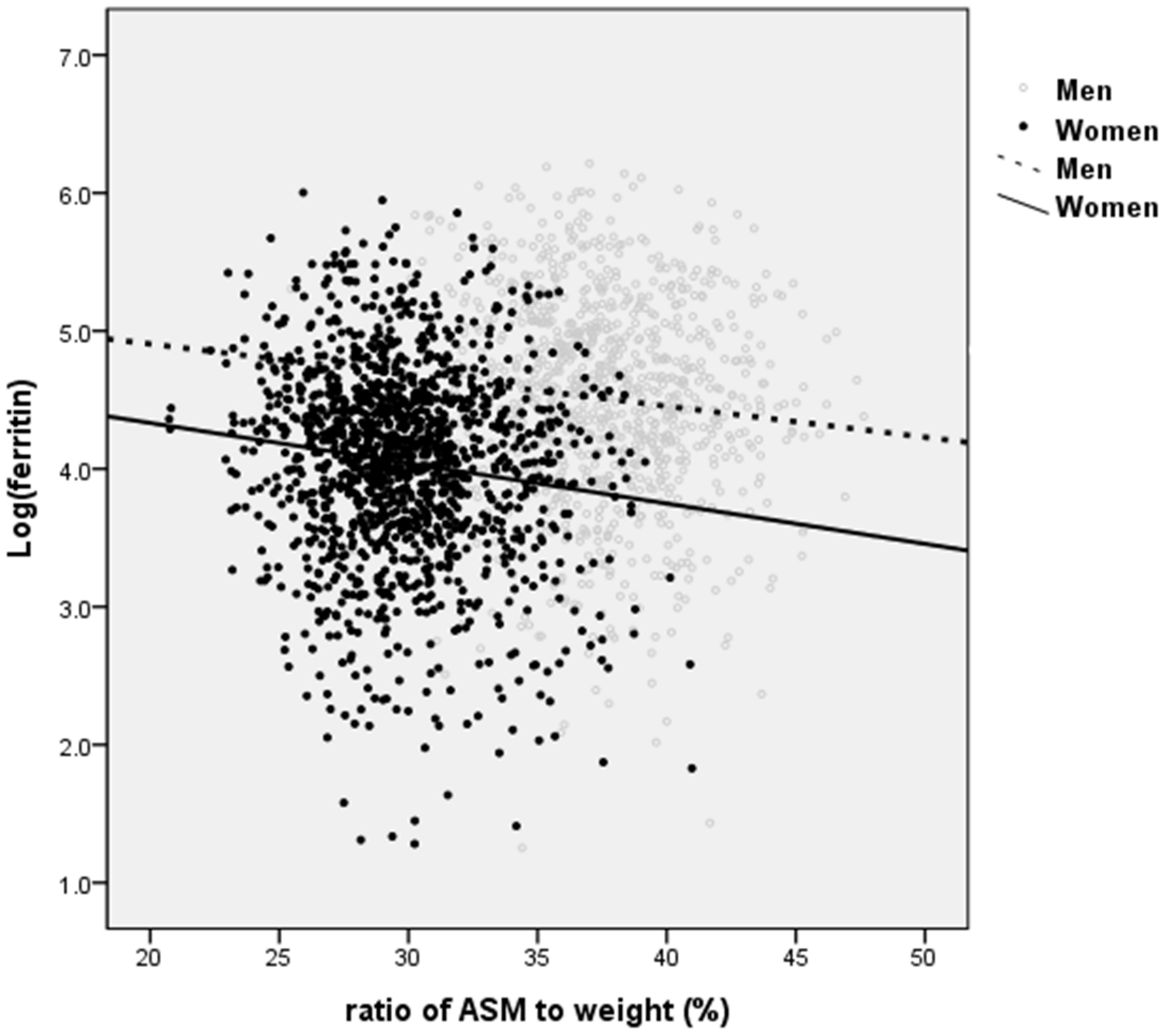
Association between ASM percent and serum ferritin (after log-transformation) in both sexes (r = −0.096, p = 0.003 in men, r = −0.126, p<0.001 in women).

Anthropometric characteristics of the subjects are provided in Table 1. The mean age was 69.0±6.3 years in men and 69.3±6.4 years in women (p = 0.354). The ASM percent was 37.6±3.2% in men and 29.8±3.1% in women (p<0.001). The proportions of subjects with very old age (>80 years; p = 0.007 in men; p = 0.002 in women), obesity (BMI>25 kg/m^2^; p<0.001 in both), and additional (>3) comorbidities (p = 0.016 in men; p<0.001 in women) were higher in men and women with sarcopenia in than those without sarcopenia. HOMA-IR was much higher in the sarcopenia group (p<0.001 in both), while the vitamin D level was lower in the sarcopenia group (p<0.001 in men, p = 0.001 in women). The serum ferritin level was much lower in women than men (men without sarcopenia 115.7 ng/mL and sarcopenia 134.4 ng/mL vs. women without sarcopenia 70.7 ng/mL and with sarcopenia 85.4 ng/mL). Furthermore, women with sarcopenia showed a significantly higher level of serum ferritin than women without sarcopenia (p = 0.001; Table 2). We observed a positive association between ASM percent and serum ferritin (after log-transformation) in men and women (r = −0.096, p = 0.003 in men, r = −0.126, p<0.001 in women; Fig. 1).

**Table 1:** Clinical characteristics of subjects.

Variables	Study population			Reference groups		
	Men (N = 952)	Women (N = 1380)	P-value	Men (N = 1001)	Women (N = 1605)	P-value
Age (years)	69.0±6.3	69.3±6.4	0.354	31.0±5.5	31.2±5.5	0.313
Anthropometrics						
Height (cm)	165.3±5.9	151.5±5.9	<0.001	173.2±5.9	160.4±5.5	<0.001
Weight (kg)	64.1±9.8	55.6±8.7	<0.001	71.4±11.1	56.8±9.5	<0.001
BMI (kg/m^2^)	23.4±3.0	24.2±3.2	<0.001	23.8±3.3	22.1±3.5	<0.001
Waist circum (cm)	85.0±9.1	83.3±9.5	<0.001	81.9±9.1	74.1±9.4	<0.001
Total fat mass (kg)	14.2±4.8	18.7±5.3	<0.001	14.9±5.9	17.7±5.6	<0.001
Total muscle mass (kg)	49.2±6.4	36.4±4.5	<0.001	55.7±6.8	38.5±4.9	<0.001
ASM (kg)	21.2±3.1	14.6±2.0	<0.001	25.2±3.5	15.9±2.4	<0.001
ASM/Height^2^ (kg/m^2^)	7.7±0.9	6.3±0.7	<0.001	8.4±0.9	6.2±0.8	<0.001
Fat percent (%)	21.9±5.2	33.4±5.5	<0.001	20.6±5.8	30.9±5.4	<0.001
ASM percent (%)	37.6±3.2	29.8±3.1	<0.001	35.5±3.1	28.2±2.6	<0.001

The data were analyzed by independent t test.

ASM: appendicular skeletal muscle. BMI: body mass index.

Data are shown as means ± the standard deviation.

**Table 2:** Clinical characteristics of Korean elders according to sarcopenia.

Variables	Men			Women		
	Without sarcopenia (N = 875)	With sarcopenia (N = 77)	P-value	Without sarcopenia (N = 1259)	With sarcopenia (N = 121)	P-value
Age (over 80 years old)	54 (6.2)	11 (14.3)	0.007	548 (43.5)	70 (57.9)	0.002
BMI (over 25 kg/m^2^)	216 (24.7)	46 (59.7)	<0.001	440 (34.9)	91 (75.2)	<0.001
Hx of Smoking	717 (81.9)	60 (77.9)	0.383	124 (9.8)	11 (9.1)	0.789
Alcohol drinking(low risk and high risk)	618 (70.6)	56 (72.7)	0.698	490 (38.9)	30 (24.8)	0.002
Marital status (coupled)	803 (91.8)	72 (93.5)	0.592	573 (45.5)	68 (56.2)	0.024
Region (urban)	497 (56.8)	49 (63.6)	0.245	721 (57.3)	77 (63.6)	0.175
Family income (low)	355 (40.6)	35 (45.5)	0.404	642 (51.0)	63 (52.1)	0.822
Education (elementary)	419 (47.9)	35 (45.5)	0.682	1041 (82.7)	104 (86.0)	0.361
Comorbidity (3 or more)	200 (22.9)	27 (35.1)	0.016	612 (48.6)	84 (69.4)	<0.001
Energy intake (above average)	448 (51.2)	28 (36.4)	0.013	642 (51.0)	48 (39.7)	0.017
Protein intake (above average)	443 (50.6)	33 (42.9)	0.191	643 (51.1)	47 (38.8)	0.010
Physical activity (above average)	444 (50.7)	33 (42.9)	0.185	640 (50.8)	50 (41.3)	0.046
WBC count	6.13±1.39	6.42±1.47	0.080	5.69±1.42	6.32±1.53	<0.001
Ferritin^a^	115.7±82.1	134.4±98.2	0.250	70.7±50.3	85.4±57.9	0.001
HOMA-IR^a^	2.25±1.45	3.16±1.76	<0.001	2.58±1.94	4.26±10.31	<0.001
Vitamin D	23.3±7.7	19.5±6.9	<0.001	20.05±7.6	17.7±6.9	0.001

Data are mean ± S.D or N (%).

a Values have been analyses after log-transformation.

### 2. Factors Associated with Sarcopenia in Older Koreans

When we used ASM percentage as the definition of sarcopenia, the prevalence of sarcopenia increased as the tertile of serum ferritin increased. However, statistical significance was only seen in elderly women (1^st^ tertile 6.3%, 2^nd^ tertile 8.0%, 3^rd^ tertile 12.0%; p = 0.008; Table 3).

**Table 3:** Prevalence of the sarcopenia according to gender-specific tertile of serum ferritin.

	*tertile of serum ferritin (ng/ml)*			
Men (n = 952)	1^st^ tertile (n = 318)	2^nd^ tertile (n = 317)	3^rd^ tertile (n = 317)	P-value
**Sarcopenia prevalence (%)**	25 (7.9)	20 (6.3)	32 (10.1)	0.213
**Women (n = 1380)**	**1^st^ tertile (n = 460)**	**2^nd^ tertile (n = 460)**	**3^rd^ tertile (n = 460)**	**P-value**
**Sarcopenia prevalence (%)**	29 (6.3)	37 (8.0)	55 (12.0)	0.008

Data are N (%). P-values were calculated by chi-square test.

Serum ferritin levels are as follows: for men-1^st^ Q (≤69.51), 2^nd^ Q (69.52–130.40), 3^rd^ Q (130.40<); for women-1^st^ Q (≤45.90), 2^nd^ Q (45.91–77.45), 3^rd^ Q (77.45<), respectively.

Spearman correlation analysis showed that ASM percentage was negatively associated with ferritin level (r = −0.111, p = 0.001 in men; r = −0.104, p<0.001 in women), BMI (r = −0.488, p<0.001 in men; r = −0.595, p<0.001 in women), waist circumference (r = −0.523, p<0.001 in men; r = −0.587, p<0.001 in women), body fat (r = −0.862, p<0.001 in men; r = −0.885, p<0.001 in women), number of comorbidities (r = −0.184, p<0.001 in men; r = −0.197, p<0.001 in women), WBC count (r = −0.182, p<0.001 in men; r = −0.180, p<0.001 in women), hemoglobin level (r = −0.107, p = 0.001 in men; r = −0.138, p<0.001 in women), and HOMA-IR (r = −0.416, p<0.001 in men; r = −0.344, p<0.001 in women), and it was positively associated with energy intake (r = 0.142, p<0.001 in men; r = 0.133, p<0.001 in women), physical activity (r = 0.174, p<0.001 in men; r = 0.136, p<0.001 in women), and vitamin D levels (r = 0.258, p<0.001 in men; r = 0.144, p<0.001 in women) in men and women (Table 4).

**Table 4:** Correlation between appendicular skeletal muscle percentages (ASM%) and clinical variables.

Variables	Men (N = 952)		Women (N = 1380)	
	r	P-value	r	P-value
Age (years)	−0.081	0.012	−0.004	0.888
Adiposity index				
Height (cm)	0.057	0.080	0.031	0.246
Weight (kg)	−0.376	<0.001	−0.487	<0.001
Appendicular skeletalmuscle (kg)	0.158	<0.001	0.176	<0.001
Total fat mass (kg)	−0.751	<0.001	−0.749	<0.001
Body mass index (kg/m^2^)	−0.488	<0.001	−0.595	<0.001
Waist circumference (cm)	−0.523	<0.001	−0.587	<0.001
Body fat (%)	−0.862	<0.001	−0.885	<0.001
Clinical parameters				
Number of comorbidity	−0.184	<0.001	−0.197	<0.001
Physical activity(MET−min/week)	0.174	<0.001	0.136	<0.001
Energy intake (Cal/day)	0.142	<0.001	0.133	<0.001
Protein intake (g/day)	0.069	0.034	0.179	0.003
Iron intake (mg/day)	0.025	0.449	0.028	0.304
HOMA-IR^b^	−0.416	<0.001	−0.344	<0.001
Vitamin D (ng/ml)	0.258	<0.001	0.144	<0.001
Ferritin (ng/mL)	−0.111	0.001	−0.104	<0.001
Hemoglobin (g/dL)	−0.107	0.001	−0.138	<0.001
WBC count (* 10^3^)	−0.182	<0.001	−0.180	<0.001

Correlation coefficients (r) and P-values were calculated using the Spearman correlation analysis.

### 3. Prevalence and Odds Ratios of Sarcopenia According to Serum Ferritin Quartiles

Logistic regression analyses were conducted to investigate the association between serum ferritin tertile and sarcopenia in the elderly population (Table 5). Men and women were divided into tertiles according to levels of serum ferritin (ng/mL; 1^st^ tertile, ≤69.51, 2^nd^ tertile, 69.52–130.40, 3^rd^ tertile, >130.40 in men: 1^st^ tertile, ≤45.90, 2^nd^ tertile, 45.91–77.45, 3^rd^ tertile, >77.45 in women). In comparison with individuals categorized in the serum ferritin 1^st^ tertile, the OR (95% CI) for sarcopenia in individuals categorized in the serum ferritin 3^rd^ tertile was 2.02 (1.26–3.23) in women, when not adjusted (p for trend = 0.009). The OR for sarcopenia was attenuated but remained statistically significant in women after adjusting for age and height (2.08 (1.30–3.34), p for trend = 0.006; Model 1). When adjusting for age and BMI (Model 2), OR for sarcopenia of individuals categorized in the serum ferritin 3^rd^ tertile was 1.85 (1.12–3.06) in women (p for trend = 0.054). In Model 3, after adjusting for age, height, BMI, comorbidities, energy intake, physical activity, hemoglobin, WBC count, vitamin D, and HOMA-IR, the OR and 95% CI for sarcopenia with respect to the 3^rd^ tertile was 1.74 (1.02–2.97) in women (p for trend = 0.124). There was no statistically significant association between sarcopenia and serum ferritin tertiles in men.

**Table 5:** Odds ratios of the sarcopenia among Korean elders according to the tertile of serum ferritin.

	1^st^ tertile	2^nd^ tertile	3^rd^ tertile	p for trend
**Men**	N = 318	N = 317	N = 317	
** Unadjusted**	1	0.79 (0.43–1.45)	1.32 (0.76–2.28)	0.218
** Model 1**	1	0.85 (0.46–1.57)	1.40 (0.80–2.43)	0.219
** Model 2**		0.77 (0.40–1.48)	0.92 (0.50–1.67)	0.726
** Model 3**	1	0.89 (0.45–1.77)	1.40 (0.75–2.65)	0.349
**Women**	N = 460	N = 460	N = 460	
** Unadjusted**	1	1.30 (0.79–2.15)	2.02 (1.26–3.23)	0.009
** Model 1**	1	1.30 (0.79–2.16)	2.08 (1.30–3.34)	0.006
** Model 2**	1	1.39 (0.81–3.21)	1.85 (1.12–3.06)	0.054
** Model 3**	1	1.36 (0.78–2.38)	1.74 (1.02–2.97)	0.124

Model 1: Adjusted for age and height.

Model 2: Adjusted for age and body mass index.

Model 3: Adjusted for age, height, BMI, comorbidity, log(energy intake), log(physical activity), hemoglobin, WBC count, Vitamin D and log(HOMA-IR).

Serum ferritin levels are as follows: for men-1^st^ tertile (≤69.51), 2^nd^ tertile (69.52–130.40), 3^rd^ tertile (130.40<); for women-1^st^ tertile (≤45.90), 2^nd^ tertile (45.91–77.45), 3^rd^ tertile (77.45<), respectively.

## Discussion

We found that higher serum ferritin levels appeared to be associated with the prevalence of sarcopenia in a nationally representative sample of older Korean women. To our knowledge, this is the first reported study to evaluate the correlation between serum ferritin levels and sarcopenia in an elderly population. However, whether the elevated serum ferritin level is a primary cause or a secondary effect of sarcopenia could not be determined from this cross-sectional study. Although the pathogenic connection between serum ferritin and sarcopenia is not understood, oxidative stress and chronic inflammation may play role in the development and progression of sarcopenia.

Iron is a transition metal that can cause oxidative tissue damage by inducing free radical formation [25], [26]. Free radicals have deleterious effects on many cellular structures, leading to cellular stress and subsequent tissue damage [26]–[28]. Merkel et al. reported that muscular iron accumulation decreased glucose uptake due to muscle damage [29]. Since Kondo et al. demonstrated the importance of iron overload in muscle atrophy by showing mitigation of oxidative damage and attenuation of muscle mass loss after iron chelation in hind limb immobilized rats [30], several studies have reported muscular iron accumulation with aging and atrophying conditions [20], [21], [31]–[33]. Iron accumulation in skeletal muscle has been suggested to contribute to the pathogenesis of sarcopenia and acute muscle atrophy [34]. Hofer et al. reported increased non-heme iron levels to 233% in rat gastrocnemius muscles with age [32]. After hind limb suspension, further elevation of non-heme iron levels in older rats, but not in young rats, were also demonstrated. In the same study, free iron accumulation was found in atrophied fibers rather than normal fibers. These results suggested a causal relation between iron overload and loss of muscle mass. Xu et al. [33] also found that total non-heme iron levels increased progressively with age in the rat gastrocnemius muscle. This increase in non-heme iron levels was also correlated with both decreased muscle mass and grip strength.

According to the mitochondrial free radical theory of aging (MFRTA), mitochondrial dysfunction due to oxidative damage to mitochondrial DNA is a major mechanism in the aging process [35]. Iron accumulation within mitochondria may enhance susceptibility to apoptosis during the development of sarcopenia, through exacerbation of oxidative stress. Iron accumulation was also found in rat skeletal muscle and liver mitochondria with aging. Mitochondrial non-heme iron levels showed positive correlations with caspase-9 and caspase-3 activities, which are activated during cell apoptosis, and total muscle non-heme iron also showed a positive correlation with the extent of apoptotic DNA fragmentation [31].

Several studies have reported associations between serum ferritin levels and metabolic disorders, including hypertension, dyslipidemia, insulin resistance, type 2 diabetes, and obesity. Some possible mechanisms for the association between serum ferritin and metabolic disorders have been proposed. Elevated body iron may contribute to insulin resistance through oxidative stress and chronic inflammation [17]. Under oxidative stress, elevated serum ferritin may promote abnormal insulin secretion by pancreatic β-cells and impairment of insulin extraction by the liver [17], [18]. Chronic oxidative stress is associated with dysfunction of β-oxidation of long chain fatty acids in the mitochondria and with β cell dysfunction in the pancreas [36], [37]. Skeletal muscle damage by iron overload is also responsible for diminished insulin-mediated glucose disposal, independent of obesity [38]. Regarding the results above, serum ferritin levels could be a predictor of sarcopenia and an important bridge between sarcopenia and metabolic disorders. Further studies are required to confirm whether elevated serum ferritin predicts sarcopenia, or whether it is merely a secondary marker of metabolic abnormalities.

In this study, the association between sarcopenia and serum ferritin levels was more prominent in elderly women than in elderly men. There are some possible explanations for this observation. Gavin et al. suggested the iron set-point theory [39] which states that healthy elderly individuals have different steady state levels of iron stores, which may be under genetic control. At steady state, iron absorption is limited to the minimal amounts necessary to cover basal iron losses. However, the age at which an individual reaches his or her iron stores set-point has not yet been determined. Garry et al. [40] suggested that this may occur at age ∼40 years in men and after menopause in women. Thus, they considered that the time was not sufficient for postmenopausal women to have reached steady-state levels of iron stores, and thus, there was a significant increase in iron stores with age in women. Consistent with this, the use of hormone replacement therapy could delay reaching steady-state iron levels, due to continued blood loss through menstruation. From set-point theory, a significant increase in iron could explain the association between iron and sarcopenia, especially in elderly women. Also, many studies have shown more prominent correlations between serum ferritin levels and metabolic disorders and higher odds ratios for metabolic disorders according to serum ferritin concentrations in elderly women than in men [12], [41]–[43].

When interpreting our results, several study limitations should be considered. First, the cross-sectional nature of this study made it impossible to assess any cause-and-effect relationships. Further prospective research is warranted to better understand causal relationships between serum ferritin levels and sarcopenia. Second, because only community-dwelling individuals were included in this study, all participants were relatively healthy. Thus, the prevalence of sarcopenia may have been underestimated. Third, serum ferritin is an acute-phase reactant and may be increased under inflammatory conditions. Even though we excluded individuals who had increased WBC counts (>10,000 cells/mL) and adjusted for WBC counts to control for confounding factors, we could not adjust for CRP as an inflammatory marker because it was not checked the KNHANES IV. Fourth, even though the European Working Group on Sarcopenia in Older People (EWGSOP) developed a practical clinical definition and diagnostic criteria for age-related sarcopenia using the presence of both low muscle mass and low muscle function (strength and performance) [44], most previous studies have used the term ‘sarcopenia’ to indicate only indicating low muscle mass. In this study, we used a definition of sarcopenia in which ASM was taken as a percentage of body weight because this showed a closer association with metabolic parameters than when defined by ASM/height^2^ [45]. Finally, because of the nature of the survey questionnaires, we could not exclude the effects of information bias.

Despite these limitations, the present study has several strengths. First, we used data from a reliable nationally representative database, including ASM, which was measured directly with DXA. Second, strict quality controls were applied to the study procedures within KNHANES.

In conclusion, we have demonstrated that elevated serum ferritin levels were associated with an increased prevalence of sarcopenia in a representative sample of older Korean women but not in older Korean men. Prospective studies are needed to understand the role of ferritin in sarcopenia and to determine whether elevated iron stores precede the development of sarcopenia and whether a threshold exists above which ferritin levels are associated with an increased risk.
